# Lower Rates of *Staphylococcus aureus* Bloodstream Infection in Patients on Hemodialysis Receiving Trimethoprim-Sulfamethoxazole Melioidosis Prophylaxis

**DOI:** 10.1093/ofid/ofae431

**Published:** 2024-07-31

**Authors:** Aliya Bryce, Sara Davison, Bart J Currie, Johanna M Birrell, Robert W Baird, Asanga Abeyaratne, Sandawana William Majoni, Teana Brewster-O’Brien, Steven Y C Tong

**Affiliations:** Department of Infectious Diseases, Royal Darwin Hospital, Darwin, Northern Territory, Australia; Department of General Medicine, Royal Melbourne Hospital, Melbourne, Victoria, Australia; Department of Infectious Diseases, Royal Darwin Hospital, Darwin, Northern Territory, Australia; Department of Infectious Diseases, Royal Darwin Hospital, Darwin, Northern Territory, Australia; Menzies School of Health Research, Charles Darwin University, Darwin, Northern Territory, Australia; Northern Territory Centre for Disease Control, NT Health, Darwin, Northern Territory, Australia; Department of Infectious Diseases, Royal Darwin Hospital, Darwin, Northern Territory, Australia; Menzies School of Health Research, Charles Darwin University, Darwin, Northern Territory, Australia; Department of Nephrology, Royal Darwin Hospital, Darwin, Northern Territory, Australia; Flinders University and Northern Territory Medical Program, Royal Darwin Hospital Campus, Darwin, Northern Territory, Australia; Menzies School of Health Research, Charles Darwin University, Darwin, Northern Territory, Australia; Department of Nephrology, Royal Darwin Hospital, Darwin, Northern Territory, Australia; Flinders University and Northern Territory Medical Program, Royal Darwin Hospital Campus, Darwin, Northern Territory, Australia; Department of Nephrology, Royal Darwin Hospital, Darwin, Northern Territory, Australia; Victorian Infectious Diseases Service, The Royal Melbourne Hospital at the Peter Doherty Institute for Infection and Immunity, Melbourne, Victoria, Australia; Department of Infectious Diseases, The University of Melbourne at the Peter Doherty Institute for Infection and Immunity, Melbourne, Victoria, Australia

**Keywords:** antibiotic prophylaxis, bloodstream infection, hemodialysis, *Staphylococcus aureus*, trimethoprim-sulfamethoxazole

## Abstract

Hemodialysis is a risk factor for *Staphylococcus aureus* bloodstream infection (SAB). In this single-center study, SAB rates were 56% lower during the monsoonal wet season when patients on hemodialysis receive supervised melioidosis prophylaxis with trimethoprim-sulfamethoxazole. This intervention may reduce SAB rates in high-risk patients; however, further targeted studies are required.

## Graphical Abstract

**Graphical abstract:**
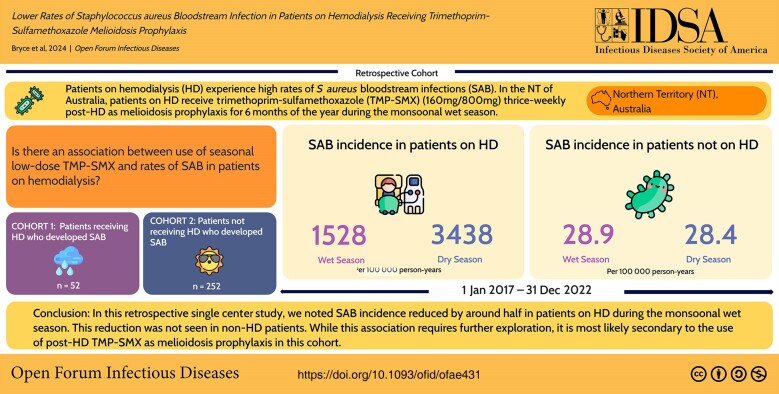
https://tidbitapp.io/institutional-portal/open-forum-infectious-diseases/tidbits/lower-rates-of-staphylococcus-aureus-bloodstream-infection-in-patients-on-hemodialysis-receiving-trimethoprim-sulfamethoxazole-melioidosis-prophylaxis/update

## Teaser

In the Northern Territory of Australia, patients on hemodialysis receive seasonal post-dialysis Trimethoprim-Sulfamethoxazole as melioidosis prophylaxis. This single-center study explored the effect of this intervention on rates of Staphylococcus Aureus bloodstream infections in this high risk group.

Trimethoprim-sulfamethoxazole (TMP-SMX) is a broad-spectrum antimicrobial used in the treatment of a number of bacterial and fungal infections. The drug is also employed in lower dosages as a prophylactic agent, primarily in immunocompromised individuals, to reduce incidence of *Pneumocystis jirovecii* pneumonia (PJP). In the tropical Top End of the Northern Territory (NT) of Australia, patients requiring maintenance renal replacement therapy with hemodialysis receive supervised postdialysis TMP-SMX (160 mg/800 mg thrice weekly) for 6 months of the year during the monsoonal wet season as prophylaxis against *Burkholderia pseudomallei*, the causative agent of melioidosis [1]. This intervention has also been associated with a decrease in incidence of invasive *Streptococcus pyogenes* (group A *Streptococcus*) infection in the target population during the period of administration [2].

Literature from the premodern combined antiretroviral therapy era indicates that prophylactic TMP-SMX may reduce community-acquired [3] and invasive *Staphylococcus aureus* infections in patients living with human immunodeficiency virus (HIV) [4]. In patients on hemodialysis, who are at significantly elevated risk of *S aureus* bloodstream infection (SAB) [5] and associated morbidity and mortality, quantifying prophylactic TMP-SMX's efficacy against SAB is of particular relevance. Here, we describe the association of wet season TMP-SMX melioidosis prophylaxis and incidence of SAB in patients receiving hemodialysis in the NT during the wet and dry seasons.

## METHODS

We conducted a retrospective cohort study in a single tertiary center, the Royal Darwin Hospital, a 360-bed hospital in the NT. The hospital and microbiology laboratory serves the population of Greater Darwin as well as remote and very remote communities across the NT. We identified blood cultures isolating *S aureus* from sterile draw in adults aged >18 years from laboratory databases from 1 January 2017 through 31 December 2022. We cross-referenced the unique medical record numbers of all patients with SAB against a local renal dialysis database to identify those concurrently receiving hemodialysis at the time of bloodstream infection and used the same database to determine the total number of patients dialyzed during the study period. We excluded any repeated episodes of SAB within 3 months from further analysis. We obtained patient demographics including sex, ethnicity, age, and comorbidities from electronic hospital reporting systems and determined cause of death from electronic death certificates.

The annual “wet season” in the NT during which prophylactic TMP-SMX (160 mg/800 mg thrice-weekly post-hemodialysis) is administered is defined as 1 November–30 April. We classified *S aureus* isolates as methicillin-resistant *S aureus* (MRSA) if cefoxitin resistant on VITEK2, and TMP-SMX resistant if VITEK minimum inhibitory concentration was ≥40 μg/mL. We calculated MRSA and TMP-SMX resistance rates from local antibiograms of *S aureus* sterile site isolates sampled 15 December 2021 to 15 June 2022. To estimate local SAB incidence, we defined a total hospital catchment population of 200 450 [6] with an estimated 26.7% of this population expected to be aged <19 years as per 2016 NT Census data (equaling 146 950 adults) [7]. We obtained a point prevalence of patients on hemodialysis from Australian and New Zealand Dialysis and Transplant Registry (ANZDATA) reporting as of 31 December 2022 [8] and used this to calculate an estimated hemodialysis SAB incidence. The χ^2^ test was used for comparison of categorical variables and Student *t* test for continuous variables. We obtained ethical approval from the Human Research Ethics Committee of the Northern Territory Department of Health and Menzies School of Health Research (NT HREC 2023-4564).

## RESULTS

A total of 1145 patients received hemodialysis in the NT over the 6-year study period. The prevalent number of hemodialysis patients on 31 December 2022 was 349 patients [8]. There were 304 SABs in adults of which 52 (17%) occurred in patients receiving hemodialysis. The estimated incidence of SAB was 2483 per 100 000 person-years in the hemodialysis population and 28.6 per 100 000 person-years in the nondialysis population. The median age of hemodialysis patients with SAB was 53.1 years (interquartile range [IQR], 46.8–61.5 years), 34 of 52 (65%) patients were female, and 39 of 52 (75%) were Aboriginal Australians. Median time on hemodialysis prior to SAB was 3.6 years (IQR, 0.6–5.8 years) noting that 9 of 52 (17%) episodes occurred within the first 3 months of commencing hemodialysis. The SAB was related to an indwelling catheter (eg, tunneled line or permacath) in 24 of 52 (46%) episodes. The *S. aureus* was MRSA in 22 of 52 isolates (42%) compared to local antibiogram MRSA rates of 596 of 1678 (35.5%) (*P* = .31). The isolate was TMP-SMX resistant in 17 of 52 isolates (33%), comprising 4 of 30 (13%) methicillin-susceptible *S. aureus* and 13 of 22 (59%) MRSA. *Staphylococcus aureus* TMP-SMX resistance rates in the local antibiogram were 148 of 1678 (8%) (*P* < .01 in comparison to hemodialysis patients). Ten patients (19%) died within 90 days of blood culture positivity; in 5 the death was attributed directly to SAB.

**Figure 1.**
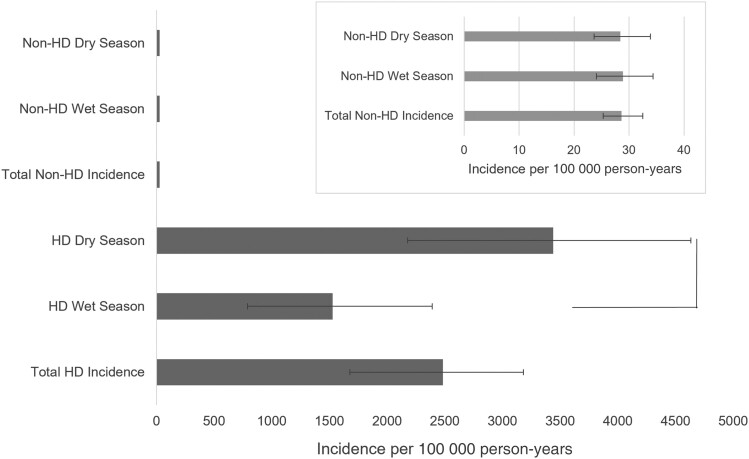
Incidence rates (per 100 000 person-years) in patients receiving or not receiving hemodialysis (HD) between the wet and dry seasons in the Northern Territory (main figure). The inset figure shows the lower rates in the non-HD population on a magnified x-axis. Error bars represent 95% confidence intervals.

Of the 52 episodes of SAB in patients receiving hemodialysis, 16 (31%) occurred during the wet season and 36 (69%) in the dry season. Wet season incidence equated to 1528 per 100 000 person-years compared with 3438 per 100 000 person-years in the dry season (incidence rate ratio, 0.44 [95% confidence interval, .23–.82]; *P* = .005). In nondialyzing adults with SAB (n = 252), there was no seasonal difference, with 50.4% (127/252) of episodes occurring during the wet season (Figure 1). Aside from the use of prophylactic TMP-SMX, there were no significant differences between recorded patient characteristics with wet or dry season SAB (Supplementary Table 1).

During the study period, 821 of all patients dialyzed (71.8%) were prescribed at least 1 dose of TMP-SMX. The majority of SAB infections in patients on hemodialysis occurred in those not receiving TMP-SMX at the time of infection (37/52 [71%]); however, there were 15 breakthrough SABs (29%) including 7 (47%) with TMP-SMX–susceptible *S. aureus* isolates. Four of the 16 patients with wet season SABs were not receiving prophylaxis with reasons of drug intolerance (n = 1), recent hemodialysis commencement (n = 1), visiting from interstate (n = 1), and unclear (n = 1). Three of the patients with dry season SABs (8%) were receiving TMP-SMX due to underlying immunosuppression at the time of bacteremia.

## DISCUSSION

This single-center retrospective study took advantage of an established treatment guideline in which patients on hemodialysis receive supervised TMP-SMX prophylaxis for 6 months of the calendar year to reduce the incidence of melioidosis during the monsoonal wet season. As the dose is supervised, rates of adherence are high, providing an opportunity to explore the effects of TMP-SMX prophylaxis on rates of SAB in the hemodialysis population. SAB incidence was lower by 56% in patients receiving hemodialysis during the wet season compared to the dry season; this variation was not evident in nondialyzing adults. The majority of SAB in patients on hemodialysis occurred in those not taking prophylactic TMP-SMX.

While the use of TMP-SMX as melioidosis prophylaxis in our center is geographically specific, these findings are potentially transferrable to other patients on hemodialysis, who as a population experience significantly elevated rates of SAB [5]. Prophylactic TMP-SMX prescribing may exert its effect through temporarily decolonizing *S. aureus* carriage in susceptible individuals, as colonization can increase susceptibility to bacteremia [9]. Reduced persistent *S. aureus* nasal colonization has been described in patients living with HIV receiving PJP prophylaxis (95% receiving TMP-SMX) [10], supporting this potential mechanism of action.

While a prophylactic antimicrobial may be attractive in reducing risk of SAB, it is not without risk. TMP-SMX resistance rates were around 3 times higher in hemodialysis *S. aureus* isolates than in local antibiograms. Adverse drug events are common with TMP-SMX [11] and can be severe. Local data from northern Australia has documented thrombocytopenia and neutropenia with TMP-SMX melioidosis hemodialysis prophylactic dosing of 160 mg/800 mg given daily, with 1 of 169 patients developing drug reaction with eosinophilia and systemic symptoms [1]. In our current study, thrice-weekly post–hemodialysis dosing appeared well tolerated with only 1 patient noted as TMP-SMX intolerant, although this was not systematically evaluated. Finally, almost half of SAB episodes in our cohort occurred in the context of an indwelling venous catheter. Appropriate line management and care remains a cornerstone of preventing SAB in patients on dialysis.

Our study has several limitations. The hemodialysis population in the NT is unique, with high proportions of Aboriginal Australians with complex comorbidities. The sample size is relatively small and data were gathered retrospectively. It is possible that unidentified confounders may have accounted for seasonal variations in rates of SAB in this cohort. We were not able to extract sufficient data to determine exact number of prescribing days per patient. Finally, the denominators used for estimated prevalence were based on single reported incidences from national reporting metrics and will not have accounted for fluctuations in local population or total numbers of those on hemodialysis.

## CONCLUSIONS

Antimicrobial prophylaxis with posthemodialysis TMP-SMX may be a potential component of a multifaceted approach in certain high-risk groups to reduce incidence of SAB. Further studies are warranted to evaluate the treatment effect, adverse effects, and impact on antimicrobial resistance of prophylactic TMP-SMX in high-risk groups for SAB.

## Supplementary Material

ofae431_Supplementary_Data

## Supplementary Data

Supplementary materials are available at *Open Forum Infectious Diseases* online. Consisting of data provided by the authors to benefit the reader, the posted materials are not copyedited and are the sole responsibility of the authors, so questions or comments should be addressed to the corresponding author.

## Notes

***Author contributions*.** A. B., S. Y. C. T., J. M. B., and B. J. C. designed the study. R. W. B., A. A., T. B., and S. W. M. assisted with data extraction. A. B. and S. D. analyzed the data and wrote the first draft. All authors reviewed and approved the final manuscript.

***Patient consent*.** Individual patient consent was not obtained for this study. The study was approved by and conformed to the standards of the Human Research Ethics Committee of the Northern Territory Department of Health and Menzies School of Health Research (NT HREC 2023-4564).
