# Supplementary material for: Lower Rates of Staphylococcus aureus Bloodstream Infection in Patients on Hemodialysis Receiving Trimethoprim-Sulfamethoxazole Melioidosis Prophylaxis
Source: Open Forum Infect Dis. 2024 Jul 31;11(8):ofae431. doi: 10.1093/ofid/ofae431 (PMC11310582; doi:10.1093/ofid/ofae431)
Supplement: ofae431_Supplementary_Data [file ofae431_supplementary_data.docx]

**Supplementary Table 1: Numbers of *S. aureus* isolates in patients on hemodialysis by year.**

|  | **Wet Season SAB** | **Dry Season SAB** | p value |
| --- | --- | --- | --- |
| Number (% total) | 16 (31%) | 36 (68%) |  |
|  |  |  |  |
| Male (%) | 4 (25%) | 14 (39%) | 0.33^¥^ |
| Age (mean, years) | 57 | 53.4 | 0.30 |
| Aboriginal Australian (%) | 12 (75%) | 26 (72%) | 0.83^¥^ |
| Time on Hemodialysis (years) | 3.16 | 3.75 | 0.57 |
| Device Associated Infection (%) | 8 (50%) | 21 (58%) | 0.58^¥^ |
| 90-day mortality (%) | 2 (13%) | 8 (22%) | 0.41^¥^ |
| Length of Stay (days) | 12.3 | 18.9 | 0.12 |
|  |  |  |  |
| Receiving Trimethoprim-Sulfamethoxazole Prophylaxis (%) | 12 (75%) | 3 (8%) | **<0.01**^¥^ |
| Trimethoprim-Sulfamethoxazole Susceptible Isolate | 10 (63%) | 25 (69%) | 0.62^¥^ |
| Methicillin Resistant *S. aureus* isolate | 7 (44%) | 15 (42%) | 0.88^¥^ |

¥ = Chi^2^. No symbol = Student’s t test.
